# A Carvacrol-Rich Essential Oil Extracted From Oregano (*Origanum vulgare* “Hot & Spicy”) Exerts Potent Antibacterial Effects Against *Staphylococcus aureus*

**DOI:** 10.3389/fmicb.2021.741861

**Published:** 2021-11-05

**Authors:** Yuanpeng Hao, Jingyi Li, Lei Shi

**Affiliations:** ^1^Key Laboratory of Plant Resources and Beijing Botanical Garden, Institute of Botany, Chinese Academy of Sciences, Beijing, China; ^2^University of Chinese Academy of Sciences, Beijing, China

**Keywords:** carvacrol, cell membrane, oregano essential oil, proteomics, *Staphylococcus aureus*

## Abstract

Oregano essential oil (OEO), as a natural antimicrobial, has gained increased interest from food researchers and manufacturers. However, a few studies have investigated its possible antibacterial effects against *Staphylococcus aureus* using the proteomic tool. The present study aimed to explore the antibacterial effect and mechanism of a carvacrol-rich OEO extracted from *Origanum vulgare* “Hot & Spicy” on the inactivation of *S. aureus*. The gas chromatography–mass spectrometry analysis of the OEO allowed the detection of 27 compounds; the major constituent was carvacrol (84.38% of total compounds). The average diameter of the inhibitory zone (DIZ) value was 29.10 mm, and the minimum inhibitory concentration (MIC) and minimum bactericidal concentration (MBC) of OEO against *S. aureus* were 0.125 and 0.25 mg/mL, respectively. The growth curve assay indicated that the OEO prolonged the lag phase of *S. aureus*. The decrease in cell viability, changes in the integrity of cell membrane, and abnormal cell morphology further reflected the cell damage of *S. aureus* caused by the OEO. In addition, a label-free proteomic analysis was applied to analyze the regulatory networks of *S. aureus* in response to 1/2 MIC OEO-treatment stress. Of the 56 differentially expressed proteins (DEPs) identified, 26 were significantly upregulated and 30 downregulated. The Kyoto Encyclopedia of Genes and Genomes pathway enrichment analysis indicated that the DEPs were mainly involved in pathways of ribosomes; valine, leucine, and isoleucine biosynthesis; and phenylalanine, tyrosine, and tryptophan biosynthesis, which suggested that the growth inhibition of *S. aureus* might be due to the disordered effect of the OEO on protein synthesis and amino acid metabolism. These findings deepened our understanding regarding *S. aureus* survival and metabolism responses to the OEO treatment and suggested that the carvacrol-rich OEO could be used in food production environments to effectively control *S. aureus.*

## Introduction

*Staphylococcus aureus* is a facultative anaerobic Gram-positive foodborne pathogen involved in human outbreaks regarded as one of the world’s leading causes of disease outbreaks related to food consumption (Jamali et al., 2015; Kang et al., 2019). *S. aureus* produces a wide variety of toxins, including staphylococcal enterotoxins, which commonly lead to staphylococcal food poisoning during the massive growth of *S. aureus* in various foods (Hait et al., 2021; Umeda et al., 2021). Moreover, *S. aureus* is ubiquitous and widely distributed in foods and food-processing environments, such as in raw meat and meat products as well as raw milk and dairy products (Wang et al., 2013; Titouche et al., 2019). The management of *S. aureus* poisoning has become increasingly difficult because of the emergence of multi-resistant strains caused by the widespread and often inappropriate use of antibiotics in livestock (Mehli et al., 2017; Zhang et al., 2020).

In the face of increasing *S. aureus* contamination, several emerging strategies for preventing and treating the contamination have been proposed, including the use of natural antimicrobial agents. Essential oils (EOs), aromatic and volatile secondary metabolites, have the potency to combat significant pathogens due to their broad-spectrum antimicrobial effects, which have been extensively evaluated in numerous food matrices, such as fruits, vegetables, meat, and milk products (Hyldgaard et al., 2012; Kang et al., 2018, 2019; Rao et al., 2019). EOs not only exert highly effective in inhibiting multidrug-resistant bacteria (Lu et al., 2018; Cui et al., 2019) but also can greatly reduce the resistance of microbes through complex mixtures of natural compounds (Lu et al., 2018). Food-grade delivery systems, including microemulsions, nanoemulsions, and liposomes, have been widely used to enhance the antimicrobial efficacy and utilization of EOs in foods (Rao et al., 2019).

Oregano, a bushy, herbaceous perennial plant native to Europe and Central Asia, is a flavoring herb widely used throughout the world to flavor various foods and processed meals, such as salads, pizza, and sausages (Morshedloo et al., 2018). EOs extracted from the leaves and inflorescence of oregano have been claimed to have numerous useful biological activities, including antioxidant, antimicrobial, anti-inflammatory, antidiabetic, and cancer-suppressing effects (Leyva-Lopez et al., 2017). Carvacrol-rich chemotypes of oregano cover most of its natural range of biological activities, which have a vast range of applications in food industries (Emrahi et al., 2021). Carvacrol [2-methyl-5-(1-methylethyl) phenol], a monoterpenic phenol, is one of the main essential oil compounds produced by oregano plants, which exhibits significant antimicrobial activity against foodborne pathogens (Miladi et al., 2016). Cui et al. (2019) reported that the antibacterial consequences of the action of oregano essential oil (OEO) could be summarized in the following ways: changes in the permeability of the cell membrane and irreversible damage to the cell membrane; inhibition of respiratory metabolism; carvacrol, as the main component of OEO, forming chimeras with DNA; and reducing the production of a Panton–Valentine Leukocidin (PVL) toxin.

Proteomics is the systematic evaluation of all proteins expressed by one particular organism in a given time (Dos Santos et al., 2016). The quantitative proteomics based on chromatographic separations coupled with mass spectrometry improves the identification of pathogenic proteins in response to the treatment of antimicrobial agents. The technology is considered a useful means to identify and characterize the differential proteins of microbes treated with natural agents, which is a key strategy to understand better the antibacterial mechanism (Tang et al., 2020). Despite some reports on the antimicrobial activity of OEO, mainly related to its effects on bacterial phenotype and physiology, the change in the protein profile of foodborne bacteria caused by an OEO is unknown or less studied. Thus, in the present study, we investigated the potential antibacterial mechanism of a carvacrol-rich OEO against *S. aureus*. On the one hand, the changes in the cell membrane of *S. aureus* after exposure to an OEO were detected using a cryo-scanning electron microscope (cryo-SEM), flow cytometry, and laser confocal microscopy. On the other hand, the label-free quantitative proteomic analysis was employed to characterize the differentially expressed proteins (DEPs) between the OEO-treated and untreated groups, which could reveal the potential functions and the biological processes involved in the anti-*S. aureus* action of OEO.

## Materials and Methods

### Plant Materials, Essential Oil Extraction, and Bacterial Strains

The aerial parts of *Origanum vulgare* “Hot & Spicy” were harvested in the full-bloom stage in Nanyang, Henan Province, at the coordinates 32°78′N, 112°57′E, 116 m of altitude, in July 2019. Then, the aerial parts were air dried under the shade for 4 weeks. Dried samples were ground to a powder before extracting the OEO. The OEO was extracted from 100 g of powdered samples in 1000 mL of distilled water by steam distillation (Clevenger apparatus) for 3 h (Baranauskienë et al., 2013). The extracted OEO was dried using anhydrous sodium sulfate and stored in an amber bottle at 4°C until use. The *S. aureus* strain CGMCC 1.4519 was obtained from the China General Microbiological Culture Collection Center (Beijing, China). The strains were stored in the Luria-Bertani (LB) broth with 25% glycerol (*v*/*v*) at –80°C. Before each experiment, the test strain was shake-cultured in LB broth for 12 h at 37°C.

### Chemical Characterization

The composition of OEO was analyzed by gas chromatography–mass spectrometry (GC-MS) (7890A-7000B, Agilent Technologies, United States) equipped with a 30 m × 250 μm × 0.25 μm HP-5MS column (Agilent Technologies). Further, 1 μL of the sample was injected at a split mode of 40:1. The injector temperature was 250°C, and the temperature programming was as follows: the temperature remained at 40°C for 2 min and was then ramped up linearly to 77°C at a rate of 8°C/min; the temperature was ramped up to 150°C at a rate of 5°C/min, and then ramped to 185°C at a rate of 3°C/min, followed by ramping to 310°C at 60°C/min. The transfer line temperature was 280°C, and helium was used as the carrier gas at a flow rate of 1.0 mL/min through the column. The MS operating parameters were as follows: ionization energy, 70 eV; ion source temperature, 230°C; quadrupole temperature, 150°C; and mass range, 40–700 u. The OEO compounds were identified by comparing the National Institute of Standards and Technology 17 library spectra and retention index (RI) values. The RI values were calculated using a series of n-alkane (C7–C40) hydrocarbons under the same conditions. The relative percentage composition of OEO components was determined based on the peak area.

### Antibacterial Activity

#### Diameter of the Inhibitory Zone Assay

The disk diffusion method was used to assess the diameter of the inhibitory zone (DIZ) of OEO on *S. aureus*. Briefly, 100 μL of *S. aureus* suspensions (approximately 10^7^ CFU/mL) were evenly spread onto LB agar plates. Sterilized antimicrobial disks were placed on test plates, and 6-μL aliquot of pure OEO was added to the 6-mm disks. Then, the plates were incubated at 37°C for 24 h (Marques et al., 2015). The DIZ value was measured using Vernier calipers (Airaj, Tsingtao, China).

#### Determination of Minimum Inhibitory Concentration and Minimum Bactericidal Concentration

The double broth dilution method described by Kang et al. (2019) was used to determine the minimum inhibitory concentration (MIC) and minimum bactericidal concentration (MBC) values. Briefly, approximately 10^7^ CFU/mL of the *S. aureus* strains were cultured in LB broths and mixed with a range of concentrations of OEO (0.0625–8 mg/mL) in test tubes. The LB without OEO was regarded as a control. Then, these tubes were incubated at 37°C for 24 h. The MIC was considered as the lowest concentration of OEO at which no visible bacterial growth was observed. An *S. aureus* suspension from each tube with no visible bacterial growth was cultured on LB agar plates. The MBC was the lowest concentration of OEO that prevented the growth of bacterial colonies on the LB agar surface.

### Effects of Oregano Essential Oil on the *S. aureus*

#### Antibacterial Curve Assay

The growth curve determination was performed as described by Kang et al. (2020), with slight modification. The OEO was dissolved in a sterile LB broth to obtain the final concentrations of 1/8MIC, 1/4MIC, 1/2MIC, MIC, and MBC. *S. aureus* CGMCC 1.4519 growth curve was determined by measuring OD_600__*nm*_ for 0 to 24 h at 1-h intervals using a Bioscreen C Automated Microbiology Growth Curve Analysis System (Oy, Finland) at 37°C in LB broth. The OD_600__*nm*_ value of LB broth without OEO and *S. aureus* was regarded as a blank control.

### Cell Membrane Integrity Assay

An *S. aureus* suspension (approximately 10^7^ CFU/mL) was treated with or without OEO at final concentrations of 0, 1/2 MIC, MIC, and MBC at 37°C for 8 h. These treated samples were washed three times with PBS and further stained with 5 μM SYTO9 and 15 μM propidium iodide (PI) (Molecular Probes, Invitrogen, France) in the dark for 15 min (Kang et al., 2020). Next, the cells were washed three times with PBS and resuspended in PBS before analysis. The cell membrane integrity was measured on a MoFlo XDP flow cytometer (Beckman Coulter, Brea, CA, United States) and a confocal laser scanning microscope (CLSM; Zeiss LSM 980 with Elyra7, Jena, Germany).

### Cryo-Scanning Electron Microscope Analysis

*Staphylococcus aureus* cells (approximately 10^7^ CFU/mL) were treated with or without an OEO (Control, 1/2 MIC, MIC, and MBC) for 8 h at 37°C. The suspensions were centrifuged at 5000 × *g* for 10 min at 4°C. The harvested cells were washed three times with PBS and finally suspended in sterile water. The morphology of *S. aureus* cells treated or untreated with the OEO was observed using a cryo-SEM (Regulus 8100, Hitachi Co., Ltd., Japan) (Wu et al., 2014).

### Label-Free Quantitative Proteomic Analysis

#### Protein Extraction, Quantification, and Digestion

The extracted OEO was added to *S. aureus* suspension [OEO final concentration was 0.0625 mg/mL (1/2 MIC)] and incubated for 8 h. *S. aureus* cultures treated without the OEO were regarded as a control. The suspensions were centrifuged at 5000 × *g* for 10 min at 4°C. The harvested cells were washed three times with PBS. The treated and untreated cultures were ground to a powder in liquid nitrogen and suspended in lysis buffer (2 M thiourea, 7 M urea, 4% SDS, 40 mM Tris-HCl) containing 1 mM PMSF, 2 mM EDTA, and 10 mM 1,4-dithiothreitol. They were then ultrasonicated on ice for 15 min (2-s ultrasonics/3-s pause). The lysate was centrifuged at 13,000 × *g* for 20 min at 4°C. The supernatant was transferred to ice-cold acetone and then centrifuged again at 13,000 × *g* for 20 min at 4°C. The precipitated pellets were collected and then solubilized in lysis buffer. The Bradford method was used for protein quantification. Proteins were diluted fivefold with 100 mM triethylammonium bicarbonate and then digested with trypsin at an enzyme–substrate ratio of 1:50 (*w*/*w*). Peptides were desalted using a Strata-X C18 pillar (Phenomenex Inc., CA, United States) and then dried using a vacuum centrifuge.

#### SDS-PAGE Analysis

The collected proteins (20 μg) for each bacterial sample were mixed with 5 × loading buffer and further boiled for 5 min. The proteins were separated on 12.5% SDS-PAGE gel (constant current 14 mA, 90 min). Protein bands were visualized by Coomassie Blue R-250 staining (Kang et al., 2018).

#### LC-MS/MS Analysis

An LC-MS/MS analysis was performed on a Thermo Orbitrap Fusion Lumos mass spectrometer (Thermo Fisher Scientific) coupled to Easy nLC-1200 nanoLC system (Thermo Fisher Scientific, Waltham, MA, United States) for 90 min. The peptides were loaded onto a reverse phase trap column (manually packed reverse phase C18 column, 100 μm × 2.5 cm, 1.9 μm particle size; 120 Å pore diameter; Dr. Maisch GmbH Inc., Germany) connected to a C18-reversed-phase analytical column (manually packed reverse phase C18 column, 150 μm × 25 cm in buffer A (0.1% formic acid) and separated with a linear gradient of buffer B (80% acetonitrile and 0.1% formic acid) at a flow rate of 500 nL/min. The mass spectrometer was operated in the positive ion mode with the following parameters: the resolution set to 120 K for MS1. In MS1, the scan range was 350–1550 *m*/*z*. The automatic gain control was 4E5, and the charge state was 2–7. In MS2, the normalized collision energy was set to 32%. Ions were broken by higher collision dissociation and then analyzed by Orbitrap with AGC targets set at 5E4.

The MS raw data for each sample were combined and searched using the MaxQuant 1.5.3.17 software for identification and quantitation analysis. The mass spectrometry proteomics data have been deposited to the ProteomeXchange Consortium via the PRIDE (Perez-Riverol et al., 2019) partner repository with the dataset identifier PXD028357.

#### Bioinformatics Analysis

Cluster 3.0^1^ and Java Treeview software^2^ were used to carry out a hierarchical clustering analysis. A heatmap was often presented as a visual aid in addition to the dendrogram. The protein sequences of the selected DEPs were locally searched using the NCBI BLAST+ client software and InterProScan to find homolog sequences. Then, Gene Ontology (GO) terms were mapped, and sequences were annotated using the software program Blast2GO. The GO annotation results were plotted using R scripts. Following annotation steps, the studied proteins were blasted against the online Kyoto Encyclopedia of Genes and Genomes (KEGG) database^3^ to retrieve their KEGG orthology identifications and were subsequently mapped to pathways in KEGG. The enrichment analysis was applied based on the Fisher exact test, considering the whole quantified proteins as the background dataset. Benjamini–Hochberg correction for multiple testing was further applied to adjust derived *P*-values. Only functional categories and pathways with *P*-values under a threshold of 0.05 were considered significant.

### Statistical Analysis

All experiments were carried out in triplicate independently. All data were presented as the mean ± standard deviation. Differences between means were tested using one-way analysis of variance and LSD test and analyzed using IBM SPSS software (version 19.0; SPSS Inc., Chicago, IL, United States). A *P*-value ≤ 0.05 indicated a significant difference.

## Results

### Chemical Composition of Oregano Essential Oil

**FIGURE 1:**
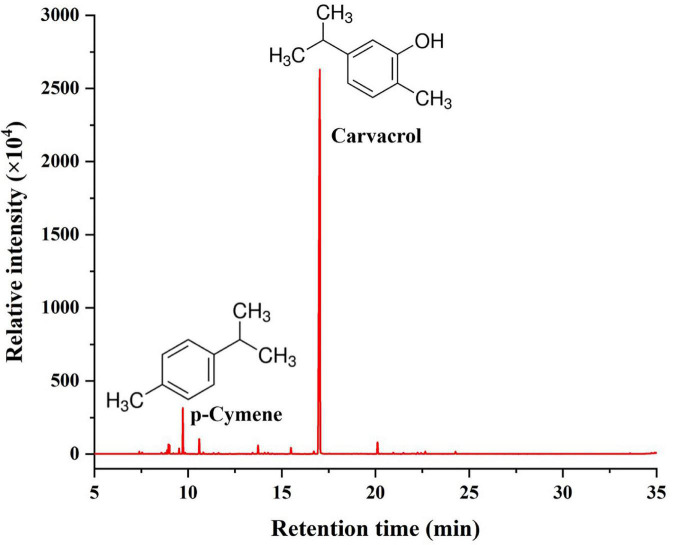
Chemical composition of OEO.

The chemical composition analysis of the OEO via GC-MS resulted in identifying 27 chemical compounds (Figure 1 and Table 1). The most abundant bioactive components were monoterpenes and sesquiterpenes. Monoterpene carvacrol was the main compound, which comprised 84.38% of the identified compounds. In addition, several other natural compounds were reported, including *p*-cymene, γ-terpinene, β-caryophyllene, and terpinen-4-ol. These compounds comprised 5.07%, 1.7%, 1.56%, and 1.06% of all compounds identified in the OEO, respectively.

**TABLE 1:** Chemical composition (%) of OEO.

**CAS**	**Compound**	**Empirical formula**	**RIref**	**RT**	**RI**	**Percentage (%)**
2867-05-2	α-Thujene	C10H16	929	7.551	932	0.420.07
123-35-3	β-Myrcene	C10H16	991	8.884	991	0.420.03
99-83-2	α-Phellandrene	C10H16	1005	9.219	1006	0.090.01
99-86-5	α-Terpinene	C10H18O	1017	9.526	1018	0.610.03
99-87-6	p-Cymene	C10H14	1025	9.722	1026	5.070.22
555-10-2	β-Phellandrene	C10H16	1031	9.835	1030	0.220.01
99-85-4	γ-Terpinene	C10H16	1060	10.595	1060	1.70.05
17699-16-0	*Trans*-Sabinene hydrate	C10H18O	1070	10.812	1069	0.310.08
586-62-9	Terpinolene	C10H16	1088	11.372	1091	0.130.01
29803-82-5	*Cis*-2-p-Menthen-1-ol	C10H18O	1126	12.234	1124	0.070.02
507-70-0	Endo-Borneol	C10H18O	1167	13.439	1170	0.170.01
562-74-3	Terpinen-4-ol	C10H18O	1177	13.738	1181	1.060.1
98-55-5	α-Terpineol	C10H18O	1189	14.083	1194	0.160.01
5948-04-9	*Trans*-Dihydrocarvone	C10H16O	1201	14.269	1201	0.250.03
6379-73-3	Carvacrol methyl ether	C11H16O	1244	15.488	1248	0.780.02
89-83-8	Thymol	C10H14O	1291	16.714	1294	0.330.05
499-75-2	Carvacrol	C10H14O	1299	16.97	1304	84.380.4
3856-25-5	Copaene	C15H24	1376	18.96	1382	0.10.05
5208-59-3	β-Bourbonene	C15H24	1384	19.232	1393	0.070.01
87-44-5	β-Caryophyllene	C15H24	1419	20.106	1428	1.560.08
6753-98-6	Humulene	C15H24	1454	20.946	1462	0.180.01
23986-74-5	Germacrene D	C15H24	1481	21.485	1484	0.380.02
3691-11-0	α-Bulnesene	C15H24	1505	21.942	1502	0.070.07
10208-80-7	α-Muurolene	C15H24	1499	22.076	1507	0.070.01
495-61-4	β-Bisabolene	C15H24	1509	22.248	1514	0.20.01
483-76-1	δ-Cadinene	C15H24	1524	22.658	1530	0.330.02
1139-30-6	Caryophyllene oxide	C15H24O	1581	24.272	1592	0.330.01

### Antibacterial Effects of Oregano Essential Oil Against *S. aureus*

Table 2 shows the DIZ, MIC, and MBC values of the OEO against *S. aureus*. As shown in Table 2, the carvacrol-rich OEO had a potent inhibitory effect against *S. aureus*. The average DIZ value was 29.1 (±0.6) mm, and the MIC and MBC of OEO against *S. aureus* were 0.125 and 0.25 mg/mL, respectively.

**TABLE 2:** Diameter of the inhibition zone (DIZ), minimum inhibitory concentration (MIC), and minimum bactericidal concentration (MBC) of OEO against *S. aureus.*

**Bacteria**	**DIZ (mm)**	**Concentrations of OEO (mg/mL)**								
		**0**	**0.0625**	**0.125**	**0.25**	**0.5**	**1**	**2**	**4**	**8**
***S. aureus***	29.1 ± 0.6	++	++	+	–	–	–	–	–	–

*“++” means observed growth of bacteria, “+” means no visible growth of bacteria, and “–” no cell colonies on the surface of LB agar.*

**FIGURE 2:**
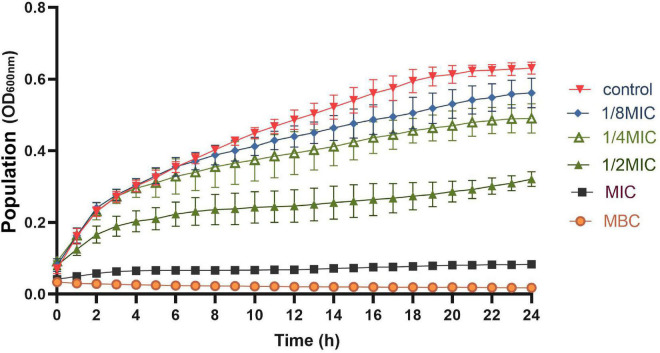
Growth curve of OEO against *S. aureus* in LB broth at 37°C; OEO concentrations includes 0 (control), 1/8MIC (0.015625 mg/mL), 1/4MIC (0.03125 mg/mL), 1/2MIC (0.0625 mg/mL), MIC (0.125 mg/mL), and MBC (0.25 mg/mL).

The effects of OEO at 1/8MIC, 1/4MIC, 1/2MIC, MIC, and MBC on the growth of *S. aureus* are shown in Figure 2. All the OEO concentrations showed inhibition against *S. aureus* but with different degrees. The growth of *S. aureus* was completely inhibited in the presence of OEO at the MIC and MBC in the LB broth. The OEO at 1/2 MIC decreased the maximal cell populations of *S. aureus* compared with the control group within 24 h. The OEO concentrations within 1/4 MIC or 1/8 MIC showed slightly inhibitory effects against the *S. aureus* growth. These results demonstrated that the concentration and treatment time of OEO had great influences on antibacterial effects.

### Effects of Oregano Essential Oil on the Cell Membrane of *S. aureus*

**FIGURE 3:**
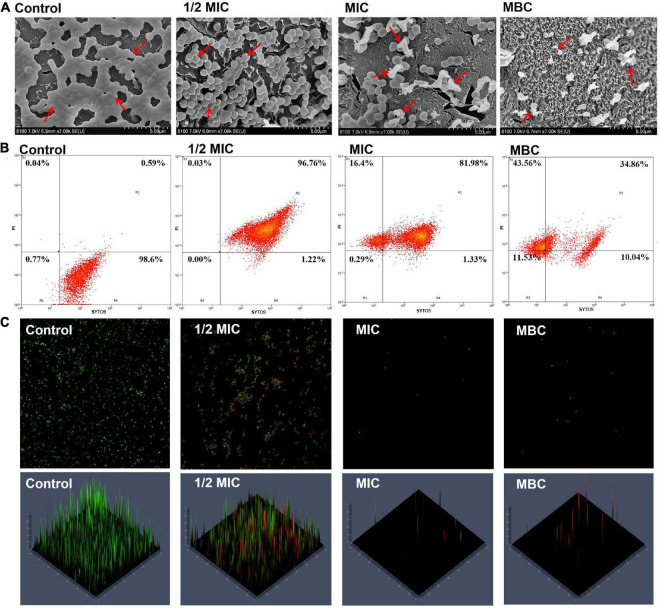
Cryo-SEM images of *S. aureus* after treatment with the OEO for 8 h at different concentrations of 1/2 MIC, MIC, and MBC **(A)**. Fluorescence dot images of *S. aureus* cells exposed to the OEO at 0, 1/2 MIC, MIC, and MBC, as analyzed by FCM **(B)**. CLSM showed the *S. aureus* viability treated with different concentrations of OEO. The cells stained with SYTO9 are labeled green, while the cells stained with PI are labeled red **(C)**.

Cryo-SEM observation allows one to study the alterations in several compartments in the cell membrane; it has been used to offer direct evidence of the ability of EOs to disrupt the structure of microbial cells (Rao et al., 2019). Figure 3A shows the changes in the cell morphology of *S. aureus.* The untreated *S. aureus* cells had a regular and smooth surface with no visible fractures and a typically spherical or elliptical *Staphylococcus* morphology. When the *S. aureus* cells were treated with OEO at 0.0625 (1/2MIC), 0.125 (MIC), and 0.25 (MBC) mg/mL, the bacterial cell surfaces showed more shrinkage compared with normal *S. aureus* cells and the number of *S. aureus* cells decreased with the increasing OEO concentration.

Furthermore, we used flow cytometry to determine the cell membrane integrity and cell viability of *S. aureus* treated with the OEO. Figure 3B shows the changes in cell viability; in this figure, R1, R2, and R4 regions correspond to dead, injured, and intact *S. aureus* cells, respectively. The control group showed approximately 98.6% *S. aureus* cells having intact cell membranes. When the *S. aureus* cells were treated with the OEO at 1/2 MIC and MIC levels, the percentage of injured cells distributed in the R2 quadrant was 96.76% and 81.98%, respectively. When the *S. aureus* cells were treated with the OEO at MIC and MBC levels, the percentage of dead cells distributed in the R1 quadrant was 16.4% and 43.56%, respectively. CLSM further verified this result. The specific performance was that the green fluorescence in the images gradually decreased and the red fluorescence gradually increased with the increase in the OEO concentration (Figure 3C).

### Protein Identification and Quantification

**FIGURE 4:**
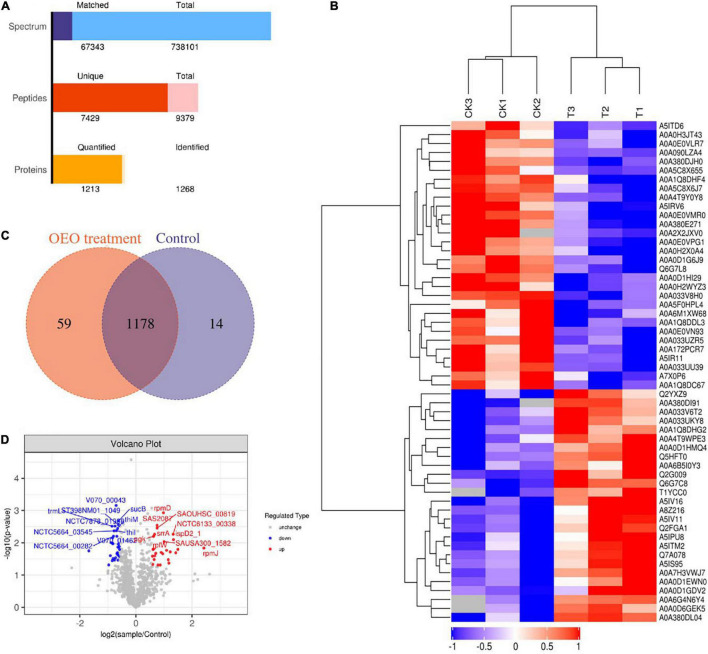
**(A)** Number of peptides that matched to proteins and number of proteins quantified and identified in this study. **(B)** Venn diagrams indicate overlapping differentially expressed proteins (DEPs) between control and OEO-treatment groups. **(C)** Volcano of DEPs. **(D)** Cluster analysis of protein expression profiles.

The label-free quantitative proteomic analysis was used to detect DEPs between control and treatment groups to explore the potential molecular mechanisms of the antibacterial effects of OEO treatment on *S. aureus*. As shown in Figure 4A, 67,343 spectra were produced and matched, and 9379 peptides were identified, of which 7429 were unique peptides. In addition, the total number of identified proteins was 1268, and the number of proteins that could be quantified was 1213. The cluster analysis of control and OEO-treatment groups is shown in Figure 4B. The result indicated only minor variations among the three replicates, but the OEO treatment differed substantially from the control.

### Distribution of Identified Differentially Expressed Proteins

A Venn diagram showed the details of all 1268 proteins between the two groups, including 1178 shared DEPs (Figure 4C). Furthermore, the volcano plot shown in Figure 4C depicts log_2_ (treatment/control) against −log_10_ (*P*-value) representing the possibility for DEPs. In this figure, DEPs were regarded as proteins with ≥2/3 or ≤2/3 fold change and *P*-value less than 0.05. As shown in Figure 4B, the red points and blue dots indicate upregulated and downregulated DEPs, respectively. The gray dots indicate unchanged proteins. Based on this, 56 DEPs were confirmed, of which 26 were significantly upregulated and 30 downregulated. Also, all the DEPs were transformed into a heat map. A hierarchical clustering analysis with the Euclidean distance algorithm was employed to analyze DEP expression patterns between control and OEO-treatment groups. As shown in Figure 4D, blue represents downregulated DEPs, red represents upregulated DEPs, and white represents no detectable expression change.

The DEPs in the control and OEO-treatment groups are listed in Table 3. DEPs with an fold change (FC) value of more than 2 included A5IV11 (50S ribosomal protein L36), A0A380DL04 (ribitol-5-phosphate cytidylyltransferase), A0A380DI91 (ribokinase), A0A033UKY8 (50S ribosomal protein L7/L12), A0A0D1EWN0 (phosphoribosylformylglycinamidine synthase subunit PurS), A5IPU8 (30S ribosomal protein S18), A8Z216 (50S ribosomal protein L33 2), and A0A1Q8DDL3 (cysteine synthase). The protein encoded by cysM in *S. aureus* was sequentially and functionally homologous to the *O*-acetylserine (thiol)-lyase B family of cysteine synthase proteins. A mutation of *cysM* caused increased *S. aureus* sensitivity to tellurite, hydrogen peroxide, acid, and diamide but decreased the ability of *S. aureus* to recover from starvation under amino acid- or phosphate-limiting conditions (Lithgow et al., 2004). Among these DEPs, A0A1Q8DDL3 protein was downregulated, suggesting that the OEO might inhibit the growth of *S. aureus* by decreasing the activity of cysteine synthase. Thus, GO and KEGG enrichment analyses were further carried to understand the biological functions regarding the inhibition of *S. aureus* by the treatment of 1/2 MIC OEO.

**TABLE 3:** Some bacterial response-related DEPs of *S. aureus* after 1/2-OEO treatment.

**Protein ID**	**Ratio**	***P*-value**	**Change**	**Protein description**
A0A033UKY8	2.33	0.019	Up	50S ribosomal protein L7/L12
A0A033UU39	0.62	0.006	Down	Glutamate dehydrogenase
A0A033UZR5	0.62	0.004	Down	Probable tRNA sulfurtransferase
A0A033V6T2	1.94	0.022	Up	Elongation factor Ts
A0A033V8H0	0.61	0.001	Down	Pyruvate dehydrogenase E1 component subunit beta
A0A090LZA4	0.51	0.049	Down	3-Deoxy-7-phosphoheptulonate synthase
A0A0D1EWN0	2.26	0.042	Up	Phosphoribosylformylglycinamidine synthase subunit PurS
A0A0D1G6J9	0.54	0.010	Down	Rqc2 homolog RqcH
A0A0D1GDV2	1.82	0.049	Up	DM13 domain-containing protein
A0A0D1HI29	0.55	0.003	Down	Putative tRNA (cytidine(34)-2-*O*)-methyltransferase
A0A0D1HMQ4	1.52	0.016	Up	Molybdopterin molybdenumtransferase
A0A0D6GEK5	1.72	0.049	Up	Esterase YdiI
A0A0E0VLR7	0.63	0.022	Down	*N*-Acyl-L-amino acid amidohydrolase
A0A0E0VMR0	0.65	0.003	Down	Transcriptional regulator, MarR family
A0A0E0VN93	0.60	0.029	Down	Coenzyme A biosynthesis bifunctional protein CoaBC
A0A0H2WYZ3	0.65	0.011	Down	NifU domain protein
A0A0H3JT43	0.59	0.034	Down	Glycine cleavage system H-like protein
A0A172PCR7	0.64	0.014	Down	Triacylglycerol lipase
A0A1Q8DC67	0.67	0.016	Down	TRAM domain-containing protein
A0A1Q8DDL3	0.31	0.018	Down	Cysteine synthase
A0A1Q8DHF4	0.66	0.026	Down	Molybdenum cofactor biosynthesis protein B
A0A1Q8DHG2	1.86	0.020	Up	Ferrichrome ABC transporter substrate-binding protein
A0A380DI91	2.51	0.005	Up	Ribokinase
A0A380DJH0	0.57	0.006	Down	Exotoxin 15
A0A380DL04	2.52	0.008	Up	Ribitol-5-phosphate cytidylyltransferase
A0A380E271	0.58	0.004	Down	Similar to hydrolase (HAD superfamily)
A0A4T9WPE3	1.50	0.026	Up	Dihydroxyacetone kinase subunit L
A0A4T9Y0Y8	0.65	0.002	Down	Dihydrolipoyllysine-residue succinyltransferase component of 2-oxoglutarate dehydrogenase complex
A0A5C8 × 655	0.57	0.031	Down	Iron-hydroxamate ABC transporter substrate-binding protein
A0A5C8 × 6J7	0.56	0.010	Down	Hyperosmolarity resistance protein Ebh (fragment)
A0A5F0HPL4	0.53	0.025	Down	Autolysin
A0A6B5I0Y3	1.51	0.046	Up	ATP-binding cassette domain-containing protein
A0A6M1XW68	0.63	0.020	Down	Recombinase RecT (fragment)
A0A7H3VWJ7	1.54	0.006	Up	Phosphoglycerate kinase
A5IPU8	2.22	0.033	Up	30S ribosomal protein S18
A5IR11	0.56	0.007	Down	Glycine cleavage system H protein
A5IRV6	0.66	0.013	Down	Phosphoribosylformylglycinamidine synthase subunit PurL
A5IS95	1.63	0.021	Up	DNA-directed RNA polymerase subunit omega
A5ITD6	0.56	0.036	Down	Uridine kinase
A5ITM2	1.57	0.033	Up	ATP-dependent 6-phosphofructokinase
A5IV11	5.36	0.015	Up	50S ribosomal protein L36
A5IV16	1.97	0.001	Up	50S ribosomal protein L30
A7 × 0P6	0.64	0.034	Down	UDP-*N*-acetylmuramoyl-L-alanyl-D-glutamate–L-lysine ligase
A8Z216	2.13	0.017	Up	50S ribosomal protein L33 2
Q2FGA1	2.00	0.009	Up	UPF0337 protein SAUSA300_1582
Q2G009	1.69	0.004	Up	Cold shock protein CspA
Q2YXZ9	1.59	0.025	Up	Probable CtpA-like serine protease
Q5HFT0	1.58	0.005	Up	Transcriptional regulatory protein SrrA
Q6G7C8	1.68	0.003	Up	Zinc-type alcohol dehydrogenase-like protein SAS2087
Q6G7L8	0.64	0.004	Down	Hydroxyethylthiazole kinase
Q7A078	1.55	0.016	Up	50S ribosomal protein L23

### Gene Ontology Functional Classification and Kyoto Encyclopedia of Genes and Genomes Pathway Analysis of Differentially Expressed Proteins

**FIGURE 5:**
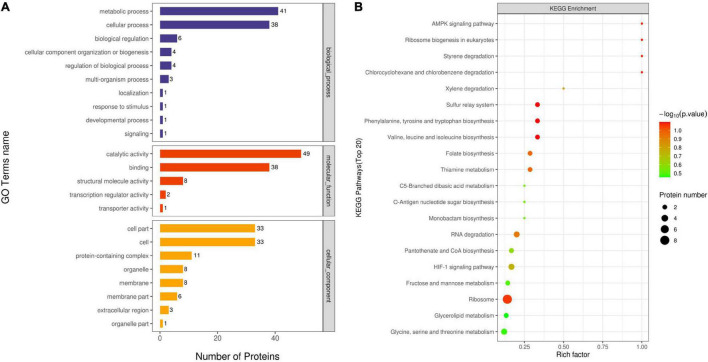
Gene ontology (GO) analysis of all DEPs **(A)**. Proteins were classified according to biological process, cellular component, and molecular function. The top terms of the KEGG pathway for DEPs for OEO treatment-vs.-control **(B)**.

A GO enrichment analysis was carried out to identify the biological processes, cellular components, and molecular functions of identified proteins. The number of proteins involved in each of these function terms is listed in Figure 5A. As illustrated in Figure 5A, metabolic process (GO:0008152) and cellular process (GO:0009987) were the main distributed terms in the biological process ontology. For the ontology of molecular function, most DEPs were mainly gathered at the following terms: catalytic activity (GO:0003824) and binding (GO:0005488). In the cellular component ontology, the main terms were cell (GO:0005623) and cell part (GO:0044464).

A KEGG pathway analysis was used to collect information regarding protein functions in the metabolic processes to explore the specific biological events of the DEPs. The distribution of the enriched KEGG pathway is shown in Figure 5B. As shown in the figure, the Rich factor represented the ratio of the number of DEPs annotated to the KEGG pathway category to the number of all identified proteins annotated to the category. The ordinate means the description of the main KEGG pathway; the bubble color indicates the significance of the enriched KEGG pathway, that is, the closer the color is to red, the smaller the *P-*value and the higher the significance level of the enrichment of the corresponding metabolic pathway. Based on this, the ribosome (ko03010) pathway enriched the largest number of DEPs and exhibited a higher significance level. Among these enriched pathways, chlorocyclohexane and chlorobenzene degradation (ko00361), styrene degradation (ko00643), ribosome biogenesis in eukaryotes (ko03008), and AMPK signaling pathway (ko04152) had a high value of the Rich factor. In addition, amino acid metabolism, including valine, leucine, and isoleucine biosynthesis (ko00290) and phenylalanine, tyrosine, and tryptophan biosynthesis (ko00400), was identified as the important enriched pathway, suggesting that the OEO might interfere with the amino acid metabolism of *S. aureus.*

## Discussion

Oregano essential oils are considered as the highly complex mixtures of bioactive compounds, in which the preponderant constituents are terpenes, generally mono- and sesquiterpenes. The main terpenes identified in the different species of oregano are carvacrol, thymol, γ-terpinene, and *p*-cymene, followed by terpinen-4-ol, linalool, β-myrcene, *trans*-sabinene hydrate, and β-caryophyllene (Leyva-Lopez et al., 2017), which was consistent with the chemical composition of OEO identified in this study. Carvacrol was present in the essential oil of *O. vulgare* subspecies: subsp. *hirtum* and subsp. *gracile* as the main components. These two subspecies were considered to be among the richest sources of EOs and carvacrol in the so-called oregano world (Emrahi et al., 2021). They suggested that under purposive or regulated water deficiency stresses, the subsp. *hirtum* yielded the higher EO content (70% higher than subsp. *gracile*), carvacrol content (20% higher), and dry material (15% higher) (Emrahi et al., 2021). Lambert et al. (2001) reported that the mixtures of carvacrol and thymol gave an additive effect, and the overall inhibition by the OEO could be attributed mainly to the additive antimicrobial action of these two compounds against *Pseudomonas aeruginosa* and *S. aureus*. Miladi et al. (2016) found that carvacrol and thymol served as the potential sources of the efflux pump inhibitor in foodborne pathogens. In addition, carvacrol can be regarded as an effective quorum-sensing inhibitor to combat the virulence and the biofilm formation of pathogenic bacteria on the surface of stainless steel (Tapia-Rodriguez et al., 2017). Therefore, exploring the antibacterial mechanism of carvacrol-rich OEO was of great significance for the further development of this natural product resource.

Previous reports confirmed the antimicrobial effect of OEO against foodborne pathogens and spoilage organisms, such as *Bacillus coagulans*, *Bacillus cereus*, *Bacillus subtilis*, *Escherichia coli*, *Pseudomonas aeruginosa*, *Vibrio cholerae*, *Listeria monocytogenes*, *S. aureus*, *Salmonella typhimurium*, and *Alicyclobacillus* spp. (Haberbeck et al., 2012; Gonçalves Cattelan et al., 2013; Dutra et al., 2019; Dogruyol et al., 2020; Das et al., 2021). These results suggested that the OEO had potential spectral antibacterial properties. Other studies suggested that the OEO exhibited the inhibition of multiple targets against drug-resistant strains and multi-drug-resistant strains, especially methicillin-resistant *S. aureus* (MRSA) (Nostro et al., 2004; Cui et al., 2019). Carvacrol at sub-inhibitory concentrations significantly inhibited the formation of mono- or dual-species biofilms by *P. fluorescens* and *S. aureus* by reducing quorum-sensing autoinductor-2 (Wang et al., 2020). Obviously, the OEO showed great advantages as an effective natural antibacterial agent to combat the antibiotic resistance and capacity for biofilm formation of foodborne pathogens.

The bacterial cell membrane is an essential active barrier structure between the cytoplasm and the extracellular medium that is important for maintaining energy transduction and cellular metabolism (Sanchez et al., 2010). Many natural agents that kill bacteria are mediated by a membrane damage mechanism. We studied the integrity of the cell membrane with the help of fluorescent probe labeling to further investigate the action mechanism of OEO on the cell membranes of *S. aureus.* PI is a fluorescent probe impermeable to cell membranes, while SYTO9 is a fluorescent probe permeable to cell membranes. The former binds to injured or dead cells and presents red fluorescence, while the latter binds to intact cells and presents green fluorescence. The two are often used in combination to assess bacterial cell membrane integrity and cell viability (Shi et al., 2016; Kang et al., 2020). Our results demonstrated that the OEO disrupted the membrane integrity of *S. aureus*. Shi et al. (2016) reported that syringic acid caused cell membrane hyperpolarization and changes in the cellular morphology of *Cronobacter sakazakii*. Kang et al. (2019) reported that the peppermint essential oil damaged the cell membranes of *S. aureus*. These finds supported the integrity results of the cell membrane damage in present study.

Proteomics analysis is a powerful technique for the identification of DEPs. Here, the label-free proteomic analysis revealed that OEO-inhibited growth of *S. aureus* might be due to the disordered effect of protein synthesis and amino acid metabolism. Based on a KEGG pathway analysis, the ribosome (ko03010) pathway enriched the largest number of DEPs. Ribosomes are organelles playing a vital role in protein synthesis. Other studies also suggest that interference with the ribosome pathway is an important means for natural products to exhibit effective antibacterial effects (Kang et al., 2020, 2021). In addition, the expression of A0A380DJH0 (exotoxin 15) protein was significantly inhibited by OEO treatment. Staphylococcal food poisoning caused by the ingestion of staphylococcal enterotoxins produced by enterotoxigenic strains of *S. aureus* has become a serious concern worldwide (Hennekinne et al., 2012). Thus, the findings on the inhibition of exotoxins by the OEO were obviously exciting. The OEO exhibited multiple targets for the inhibition of *S. aureus* according to the number and functional distribution of DEPs. Hua et al. (2018) reported that the inhibition of ribosome formation was the main mechanism of aspidinol killing *S. aureus*; the inhibition of amino acid synthesis and the reduction of virulence factors played a secondary role. Disordering the amino acid metabolism was considered a main antibacterial mechanism of OEO against *S. aureus*. Tang et al. (2020) indicated that the *Amomum villosum* Lour. essential oil affected the carbohydrate and amino acid metabolism in MRSA. The *Blumea balsamifera* (L.) essential oil disordered amino acid metabolism, physiological function and inhibited the synthesis of nucleic acids and proteins, of *S. aureus* (Yang et al., 2021). Overall, the present study provided some exciting results regarding the potent bactericidal effect of a carvacrol-rich OEO and its possible mechanisms of action. The OEO showed great potential in causing metabolic disorders, especially affecting bacterial protein synthesis and amino acid metabolism.

Sarengaowa et al. (2019) found that the DEPs generated from *L. monocytogenes* treated without and with thyme essential oil were mainly related to cellular processes, environmental information processing, genetic information processing, human diseases, metabolism, organismal systems. In accordance with the treated and untreated *Salmonella* Enteritidis, Barbosa et al. (2020) pointed out that the DEPs belonged to four distinct categories by GO (molecular function, biological process, cellular component, and protein class) and regulatory activity with greater change in expression in the *Origanum vulgare* essential oil, carvacrol, and thymol treatments. Tang et al. (2020) found that 48% of proteins were related to catalytic activity and 34% were related to binding; also, these DEPs were ranked from high to low and were related to the cellular process (32%), metabolic process (30%), and localization (18%), by the GO enrichment analysis of DEGs generated by control and *Amomum villosum* Lour. essential oil-treated MRSA groups. These results were similar to the result obtained in this study, indicating that the inhibitory effect of OEO on *S. aureus* might be closely related to the terms such as catalytic activity and binding.

## Conclusion

In conclusion, a carvacrol-rich OEO extracted from *O. vulgare* “Hot & Spicy” demonstrated potent antimicrobial activity against *S. aureus*. The OEO appeared to some intuitive mechanisms of action: decreased maximal bacterial populations, decreased bacterial viability, and damage to the cell membrane. The label-free quantitative proteome analysis was used as a powerful technique to explore further the changes in the protein expression of *S. aureus* induced by the OEO treatment. Several identified DEPs were associated with antibacterial mechanisms related to interference on the pathway of ribosome and amino acid metabolism, which broadened our understanding of the molecular mechanisms underlying the response of *S. aureus* under the carvacrol-rich OEO stress. Future studies should investigate the bactericidal effect of the OEO on *S. aureus* in different food matrices and make it more applicable through nanotechnology.

## Data Availability Statement

The mass spectrometry proteomics data have been deposited to the ProteomeXchange Consortium via the PRIDE partner repository with the dataset identifier PXD028357 (http://www.ebi.ac.uk/pride; username: reviewer_pxd028357@ebi.ac.uk; password: uqrrJEuD).

## Author Contributions

YH analyzed the data, wrote the manuscript, and performed the experiments. JL proofread the revised manuscripts. LS supervised the experiments and provided financial support of this study. All authors contributed to manuscript revision and approved the submitted version.

## Conflict of Interest

The authors declare that the research was conducted in the absence of any commercial or financial relationships that could be construed as a potential conflict of interest.

## Publisher’s Note

All claims expressed in this article are solely those of the authors and do not necessarily represent those of their affiliated organizations, or those of the publisher, the editors and the reviewers. Any product that may be evaluated in this article, or claim that may be made by its manufacturer, is not guaranteed or endorsed by the publisher.

## Footnotes

1
http://bonsai.hgc.jp/~mdehoon/software/cluster/software.htm

2
http://jtreeview.sourceforge.net

3
http://geneontology.org/
